# Increased Exercise Tolerance in G6PD African Variant Mice Driven by Metabolic Adaptations and Erythrophagocytosis

**DOI:** 10.3390/antiox14080927

**Published:** 2025-07-29

**Authors:** Francesca I. Cendali, Abby L. Grier, Christina Lisk, Monika Dzieciatkowska, Zachary Haiman, Julie A. Reisz, Julie Harral, Daniel Stephenson, Ariel M. Hay, Eric P. Wartchow, Paul W. Buehler, Kirk C. Hansen, Travis Nemkov, James C. Zimring, David C. Irwin, Angelo D’Alessandro

**Affiliations:** 1 Department of Biochemistry and Molecular Genetics, University of Colorado, Anschutz Medical Campus, Aurora, CO 80045, USA; francesca.cendali@cuanschutz.edu (F.I.C.); alg180@case.edu (A.L.G.); monika.dzieciatkowska@cuanschutz.edu (M.D.); julie.haines@cuanschutz.edu (J.A.R.); daniel.stephenson@cuanschutz.edu (D.S.); kirk.hansen@cuanschutz.edu (K.C.H.); travis.nemkov@cuanschutz.edu (T.N.); 2 Cardiovascular and Pulmonary Research Laboratory, Department of Medicine, University of Colorado, Anschutz Medical Campus, Aurora, CO 80045, USA; christina.lisk@cuanschutz.edu (C.L.); david.irwin@cuanschutz.edu (D.C.I.); 3 Department of Medicine, University of Colorado Denver, Aurora, CO 80045, USA; julie.harral@cuanschutz.edu; 4 Department of Pathology, University of Virginia School of Medicine, Charlottesville, VA 22903, USA; ah6ra@virginia.edu (A.M.H.); pbuehler@som.umaryland.edu (P.W.B.); jcz2k@virginia.edu (J.C.Z.); 5 Department of Pathology, Children’s Hospital Colorado, Aurora, CO 80045, USA; eric.wartchow@cuanschutz.edu; 6 The Center for Blood Oxygen Transport, Department of Pediatrics, University of Maryland School of Medicine, Baltimore, MD 21201, USA

**Keywords:** critical speed, exercise, G6PD, hemolytic disorder, metabolism

## Abstract

Glucose-6-phosphate dehydrogenase (G6PD) deficiency, the most common enzymatic disorder, affects over 500 million people worldwide and is often linked to exercise intolerance due to oxidative stress, but its true impact on physical performance remains unclear. This study aimed to evaluate the physiological and metabolic effects of G6PD deficiency on endurance capacity. Using humanized mice carrying the African G6PD variant [V68M; N126D] (hG6PD_A−_), we show that despite reduced pentose phosphate pathway activity, these mice exhibit a 10.8% increase in treadmill critical speed (CS)—suggesting enhanced endurance capacity. Multi-omics profiling across red blood cells, plasma, skeletal muscle, spleen, kidney, and liver reveals metabolic adaptations, including elevated glycolysis, fatty acid oxidation, and increased mitochondrial activity, alongside heightened oxidative phosphorylation in muscle and accelerated red blood cell turnover in the spleen and liver. These findings indicate that systemic metabolic reprogramming may offset antioxidant deficiencies, potentially conferring a performance advantage. Given that G6PD deficiency affects up to 13% of African Americans and is associated with cardiovascular health disparities, our results challenge conventional exercise restrictions and highlight the need for personalized exercise guidelines for affected individuals.

## 1. Introduction

Glucose 6-phosphate dehydrogenase (G6PD) is the rate-limiting enzyme of the pentose phosphate pathway (PPP), which generates the majority of cellular nicotinamide adenine dinucleotide phosphate (NADPH) [1]. NADPH-dependent reactions regulate the regeneration of reduced glutathione, ferric iron reduction to the ferrous state [2], and, directly or indirectly, NADPH/NADP^+^ ratios preserve the activity of virtually all antioxidant systems via glutaredoxins or NAD(P)H-dependent methemoglobin reductases [3,4]. G6PD activity plays an especially critical role in red blood cells (RBCs), the most numerous host cell in humans (~83% of the total), which are exposed to extensive oxidant stress during their 120 day circulatory lifespan [5]. Mature RBCs lack mitochondria and are consequently deprived of most enzymatic pathways for alternative NADPH production, rendering them highly reliant on the PPP to maintain redox balance. As such, the PPP plays a critical role in protecting RBCs from oxidative stress-induced hemolysis. When exposed to unopposed oxidative stress, cell membrane disruption renders the RBCs susceptible to intravascular hemolysis [6,7]. Furthermore, hemoglobin is denatured, forming pathogenic cytoplasmic structures known as Heinz bodies that bind to the RBC membrane [8]. These molecular lesions target the RBCs for splenic or hepatic sequestration and clearance by macrophages, a process known as erythrophagocytosis wherein damaged or senescent RBCs are engulfed and degraded by phagocytic cells of the reticuloendothelial system [9,10]. This form of clearance is referred to as extravascular hemolysis [1,6].

Millions of individuals worldwide engage in some form of daily exercise that ranges from mild to rigorous. Higher exercise capacity has been shown to be protective against early mortality [11,12,13]. Interestingly, G6PD overexpression is associated with extended lifespans in mice [14,15,16]. While acknowledging variability in exercise duration and intensity, there is broad consensus in the literature that strenuous exercise induces elevated production of reactive oxygen species (ROS) [17,18,19]. Oxidative and mechanical stress arising during exercise damages cells throughout the body, especially RBCs as they face shear stress [20] while navigating the circulatory system during exercise-induced elevation in heart rates and circulatory transit events [21]. The rheological properties of RBC membranes, including integrity and deformability [22], are negatively affected by intense exercise activity, even in recreational and professional elite athletes [19,21]. On the other hand, regular moderate exercise has been shown to enhance the antioxidant defense system, thereby improving red blood cell (RBC) resilience to oxidative stress [17,23]. Consequently, while acute, high-intensity exercise may exacerbate oxidative stress and RBC damage, consistent moderate activity can promote a protective adaptive response by balancing ROS production with antioxidant capacity [24,25].

G6PD deficiency (G6PDd) is the most common enzymatic deficiency in humans, affecting approximately 500 million people worldwide [26]. Since G6PD is encoded by a gene on the X chromosome, thus G6PDd is more prevalent in males [27]. Studies on female heterozygous carriers suggest that G6PDd traits may confer some degree of protection against the most lethal variant of malaria, *Plasmodium falciparum*, and thus may have been favorably selected for in areas where malaria is or was endemic [28]. Among the over 230 G6PDd variants, the African variant (V68M N126D), a class III moderate deficient variant that retains 10–60% residual enzymatic activity [29], is the most common in the United States, affecting 1 in 10 Black men in the U.S. [1]. The prevalence of G6PDd in these populations is not only shaped by evolutionary pressure, but also contributes to significant health disparities. Individuals with G6PDd, particularly those of African descent, face increased risks of oxidative stress-related complications that may exacerbate chronic diseases such as cardiovascular disease [30,31]. African Americans experience higher rates of heart failure, hypertension, and stroke compared to non-Hispanic whites [32]. This intersection highlights the need to explore the molecular mechanisms linking G6PDd and cardiometabolic fitness, particularly in populations with elevated prevalence of both.

Hemolytic anemia following oxidant exposure is a common and clinically relevant complication in patients with G6PDd, occurring with use of quinone antimalarials, sulfa drugs, or even consumption of fava beans [5,33,34,35]. Furthermore, increased rates of hypertension and idiopathic cardiomyopathies have been reported in individuals with G6PD deficiency [36]. In light of these observations, current recommendations advise individuals with G6PDd to avoid strenuous physical activity due to the heightened risk of exercise-induced hemolytic events, while still promoting regular physical activity to support long-term health and longevity [37]. Despite these longstanding guidelines, clinical evidence supporting these recommendations remains limited, and the molecular consequences of exercise capacity in individuals with the most severe forms of G6PDd are not well understood [38].

Exercise capacity has not been assessed mechanistically in G6PDd patients carrying the African variant owing to ethical and technical limitations [1]. Instead, mouse models of G6PDd offer a valuable platform to test this hypothesis in a tractable system via comparison to mice expressing the human form of G6PD. Recently, we investigated exercise tolerance in mice expressing the Mediterranean (S188F) G6PDd variant, one of the most severe class II variants (<10% residual activity) [39]. We unexpectedly observed increased exercise tolerance in these mice, a phenomenon at least in part explained by cardiovascular adaptations such as increased cardiac output, stroke volume, sarcomere length, and mitochondrial content [39]. However, studies on the most common G6PD variant in the United States, the African (V68M N126D) variant, have not yet been performed and are the focus of the present study. Here, we leveraged the G6PD African variant mouse model to investigate the impact of exercise in this context and assess its broader implications for health disparities.

## 2. Materials and Methods

### 2.1. G6PDd Mouse Model

The humanized mouse models are described in more detail [40,41]. Briefly, C57BL/6J mice were first engineered to express a human canonical G6PD, i.e., non-deficient (hereafter referred to as G6PD_ND_). These mice were further engineered to delete 13 residual base pairs from CRE excision of mouse G6PD. The African V68M and N126D (henceforth G6PD_A−_) was introduced via CRISPR Cas9 of G6PD_ND_ mouse embryos at the University of Virgina, Zimring Lab. All experiments were carried out under an approved institutional animal care and use committee protocol at the University of Colorado Anschutz Medical Campus (IACUC protocol n 218). G6PD activity was quantified using the G6PD Reagent Set (Pointe Scientific (Canton, MI, USA), catalog G7583180) following the manufacturer’s protocol, unless noted otherwise. The mice used within this study were two–three months of age.

### 2.2. Treadmill Exercise and Constant Speed Tests

Before critical speed (CS) determination, mice underwent treadmill familiarization with four ~5 min runs (Exer 3/6, Columbus Instruments, Columbus, OH, USA) at 10–15 m/min on a 5% incline. In later runs, the speed was increased to ~30–35 m/min to acclimate mice to high-speed running. Compressed air puffs were used for motivation. Protocols followed APS guidelines and were conducted by trained staff. This is further detailed in Supplemental Methods [42,43].

### 2.3. Data Modeling for the Determination of CS

After completing constant speed treadmill tests, CS and finite distance capacity (D′) were calculated using the linear 1/time model (Speed = D′ × 1/time + CS), where the y-intercept represents CS and the slope represents D′, as previously described [44].

### 2.4. Hemodynamics

After CS testing, terminal open chest right ventricular (RV) and left ventricular (LV) function measurements were assessed with a 1.2F, FTE-1212B-4018 pressure volume catheter (Transonic Systems Inc., Ithaca, NY, USA) as detailed in the Supplemental Methods [44].

### 2.5. Mass Spectrometry-Based Proteomics

Whole frozen tissues (~10 mg, gastrocnemius, soleus, liver, kidney, and spleen) were powdered in liquid nitrogen, lyophilized, and homogenized (~8 mg) in a high-salt buffer (50 mM Tris-HCl, 3 M NaCl, 25 mM EDTA, 0.25% CHAPS, pH 7.5) with protease inhibitors (Halt, Thermo Scientific, Waltham, MA, USA) at 10 mg/mL using a Bullet Blender (1 mm glass beads, 3 min, 4 °C). Further, mass spectrometry-based proteomics were performed as previously described and in extended detail in the Supplemental Methods [39].

### 2.6. Mass Spectrometry-Based Metabolomics and Lipidomics

Plasma and RBCs were processed using 10 uL (1:10) and whole tissues (gastrocnemius, soleus, liver, kidney, and spleen) were powderized with a mortar and pestle in a liquid nitrogen bath, then extracted at 15 mg/mL with cold MeOH:MeCN:H_2_O (5:3:2, v:v:v). Further, mass spectrometry-based metabolomics and lipidomics were performed as previously described, and the procedures are detailed in the Supplemental Methods [45].

### 2.7. Mass Spectrometry-Based Metal Analysis and Inductively Coupled Plasma Mass Spectrometry Instrumentation

ICP-MS was performed on isolated RBCs collected before and after exercise from each model as previously described, and the procedures are detailed in the Supplemental Methods [46].

### 2.8. Macrophage Isolation and Erythrophagocytosis

Spleen and livers were harvested, finely minced, and placed in 2 mL Eppendorf tubes containing 1 mL of DMEM with Collagenase D (Sigma Aldrich (St. Louis, MO, USA), product #11088866001). Details for isolation and erythrophagocytosis are further explained in detail in the Supplemental Methods [47].

### 2.9. Scanning Electron Microscopy

Whole blood samples were prepared for scanning electron microscopy (SEM) according to standard procedures and are described in detail in the Supplemental Methods [48].

### 2.10. Statistics and Modeling

Data are presented as mean ± SEM. Statistical analyses were conducted using GraphPad Prism v10.3 (San Diego, CA, USA), OmicsNet, and custom R scripts (version 4.1.0; 2023-06-16 ucrt), as previously described [43]. Heatmaps, metabolic pathway analyses, partial least squares-discriminant analysis (PLS-DA), and hierarchical clustering were generated using MetaboAnalyst 6.0. Comparisons between two groups were made using two-tailed Student’s *t*-tests with FDR adjustment, and *p* values were calculated using the log-rank test. Pathway diagrams were created using BioRender.com.

The human RBC genome-scale metabolic reconstruction (RBC-GEM version 1.2.0) [49] and the vertebrate homology data from Mouse Genome Informatics database (MGI version 6.24) [50] were utilized to create a mouse-specific RBC-GEM by mapping human genes to their mouse orthologs. Following successful acquisition of proteomic data for RBCs from hG6PD_ND_ and hG6PD_A−_ mice pre-exercise, protein copy numbers were computed via the “total protein approach” [51,52] using a mean corpuscular hemoglobin value of 13.9 picograms. Proteome-constrained models for each sample were subsequently derived following the OVERLAY workflow as previously described in detail [49,53], with samples scaled assuming hemoglobin comprised 95% of the proteome dry weight. Individual protein constraints were relaxed by 3%, and the global relaxation constraint was minimized to its smallest possible value that allowed for solution feasibility in simulations.

Proteome-constrained models were simulated using flux balance/flux variability analysis to determine maximum fluxes, maximum enzyme abundances, and effective flux ranges. Spearman rank correlation coefficients (ρ) were computed between maximum flux and enzyme abundance to classify reactions as abundance-dependent (ρ ≥ 0.8), abundance-correlated (0.5 ≤ ρ < 0.8), or abundance-independent (ρ < 0.5). Mann–Whitney U tests were conducted to determine statistically significant (*p* < 0.05) differences in flux ranges between G6PD phenotypes. All simulations performed were implemented in Python 3.11 using the COBRApy package (version 0.29.1) [54,55] and its implementation of algorithms for FBA/FVA [56]. Statistical analyses of simulation results were performed using the SciPy package (version 1.15.3) [57]. Statistically significant differences in flux ranges between G6PD phenotypes were visualized as a heatmap using the Morpheus online software tool (https://software.broadinstitute.org/morpheus, access date: 1 July 2025, Version 1.0.18). Hierarchical clustering of columns within phenotype groups was determined by Jaccard distance. Area plots of flux ranges and min–max normalized enzyme abundances across G6PD phenotypes were visualized using Matplotlib (version 3.10.3) [58].

## 3. Results

### 3.1. G6PD_A_ Mice Maintain Higher CS

**Figure 1:**
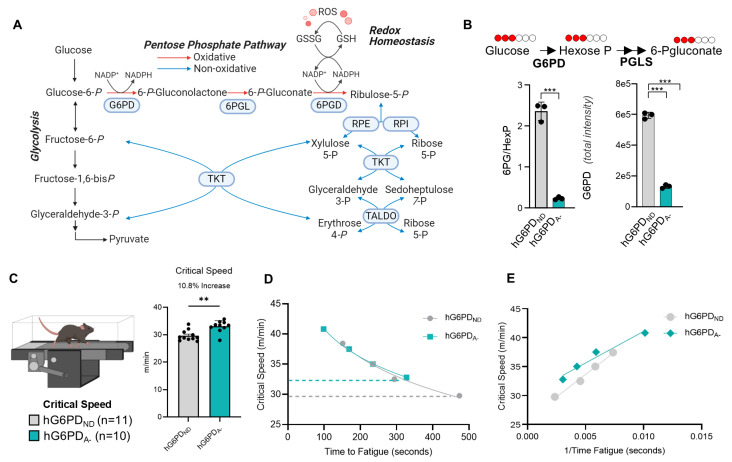
Critical speed (CS) assessment of novel G6PDd mice. (**A**) A pathway map of glycolysis and oxidative and non-oxidative metabolism in the pentose phosphate pathway (Figure created with BioRender.com). (**B**) 1,2,3-^13^C_3_-glucose tracing. (**C**,**D**) Mice run using CS method; G6PD_A_ mice maintained a significantly faster CS (10.8% increase), as measured by the *t*-test, and dashed lines indicate CS. (**E**) G6PD_A_ higher anaerobiotic work capacity (AWC) as measured by the slope, hG6PD_ND_ = 1715 and hG6PD_A−_ = 1095 (Significance **: *p* < 0.01, ***: *p* < 0.001).

To understand how G6PDd alters exercise capacity, we leveraged a recently described humanized mouse model coding for the V68M and N126D G6PD variant (hG6PD_A−_). We characterized G6PDd mice at baseline by profiling blood glucose metabolism, focusing on the pentose phosphate pathway (PPP). G6PD catalyzes the first and rate-limiting step of the oxidative PPP, converting glucose 6-phosphate to 6-phosphogluconolactone and subsequently to 6-phosphogluconate. (Figure 1A). Tracing experiments with mice were performed to monitor glucose fluxes through glycolysis and the PPP, as determined via the ratios of the 1,2,3-^13^C_3_ isotopologues of 6-Phosphogluconate/Hexose Phosphate [59]. These results further confirmed the defect in PPP activation in the hG6PD_A−_ mice. Confirming the effect of the genetic intervention, hG6PD_A−_ mice display deficient G6PD activity, as gleaned by overall reduced G6PD intensity (Figure 1B).

Next, we calculated the CS of hG6PD_ND_ and hG6PD_A−_ mice to determine exercise tolerance (Figure 1C). Unexpectedly, hG6PD_A−_ mice showed ~10.8% faster CS compared to mice carrying the canonical human G6PD (*p* < 0.02, Figure 1C,D and Supplemental Table S1). The hG6PD_A−_ mice also showed a decrease in their anaerobic work capacity (AWC), highlighting an ability to work closer to their VO_2max_ (Figure 1E). These results are consistent with observations in humanized G6PDd mice expressing the Mediterranean variant [39]. However, while hemodynamics testing previously highlighted adaptations in heart size, cardiac output, stroke volume [39]—all elevated in mice expressing the severe Mediterranean G6PD variant compared to canonical mice—here we did not observe any significant difference in cardiac function when comparing hG6PD_ND_ and hG6PD_A−_ mice (Supplementary Figure S1).

### 3.2. Pre- and Post-Exercise Blood Analysis

**Figure 2:**
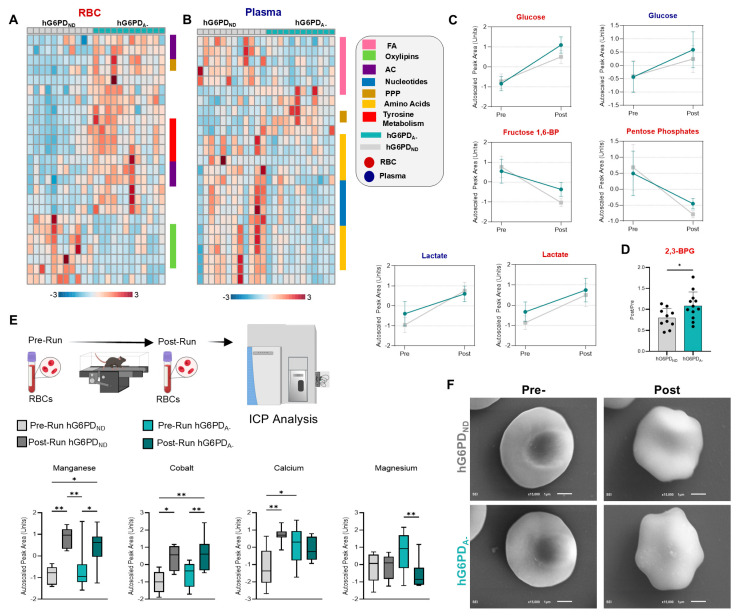
Metabolic alterations detected in blood pre- and post-exercise. (**A**) RBC and (**B**) plasma heat maps of top 25 metabolites significantly different (ANOVA) between hG6PD_ND_ and hG6PD_A−_ taken from the post/pre-fold-change (AC = acylcarnitines, FA = fatty acids). (**C**) Glycolysis, pentose phosphate pathway, and TCA intermediates before and after CS tests. Metabolite name is color coded according to fraction: plasma (blue) and RBC (red) and lines coded according to G6PD status hG6PD_ND_ (gray) and hG6PD_A−_ (aqua). (**D**) Post/pre-fold-change of 2,3-BPG. (**E**) Trace metal analysis of RBCs before and after exercise. (**F**) Scanning electron microscopy images of RBC morphology pre- and post-run to exhaustion (Significance: *: *p* < 0.05, **: *p* < 0.01).

The unexpected finding of increased CS in hG6PD_A−_ mice and lack of cardiac function phenotype could explain a potential adaptation, suggesting that alternative mechanisms underlie increased tolerance. Specifically, we hypothesized that genetic perturbation of fluxes through the PPP may favor a metabolic reprogramming in mature RBC towards glycolysis and the Rapoport–Luebering shunt. Indeed, as oxygen availability is critical to exercise tolerance by fueling mitochondrial function, metabolic adaptations in RBCs—such as elevation in 2,3-bisphosphoglycerate (BPG)—would contribute to RBC oxygen off-loading by promoting hemoglobin allostery via the stabilization of its tense deoxygenated state [60,61,62]. This adaptation is commonly observed in response to hypoxia [63,64], for example, upon exposure to high altitude [65,66]. To test this hypothesis, metabolomics analyses of RBCs and plasma from hG6PD_ND_ and hG6PD_A−_ mice, collected both pre- and post-run, were performed to determine the impact of exercise on RBC and plasma metabolism. First, post/pre-exercise fold-change ratios of metabolites highlighted differences in RBCs with respect to G6PD status. Oxylipins decreased while non-oxidative PPP and acylcarnitine (AC) intermediates increased in RBCs from hG6PD_A−_ mice compared to hG6PD_ND_ (Figure 2A and details elaborated in Supplemental Figure S2A). Plasma metabolites show decreases in the post/pre-exercise ratio of nucleosides and amino acids in G6PD_A_ compared to hG6PD_ND_ (Figure 2B and details elaborated in Supplemental Figure S2B). Focusing on glycolytic metabolites, we see little difference across genotypes in RBC and plasma levels of glucose and fructose 1,6-bisphosphate, and no difference in levels of lactate (Figure 2C). Consistent with the G6PDd state, baseline levels of PPP metabolites were lower in the hG6PD_A−_ mice, though a significant post-exercise drop in PPP end-product pentose phosphate was observed in hG6PD_ND_ compared to hG6PD_A−_, suggestive of a preference towards glycolytic fluxes rather than the PPP in response to exercise. Consistent with our hypothesis, we observed a significant increase in BPG (Figure 2D) in hG6PD_A−_ compared to hG6PD_ND_ mouse RBCs in response to exercise. Plasma analysis revealed no significant differences in key hemolysis markers, including metabolic indicators (biliverdin, bilirubin, and heme) and proteomic markers (Hba and Hbb-b1), between post- and pre-exercise states (Supplemental Figure S2C). Pathway enrichment analysis of the significant post/pre metabolite ratios in G6PD_A−_ RBCs revealed enrichment in tyrosine, arginine, and glutathione metabolism. Similarly, plasma analysis highlighted significant enrichment in glutathione and arginine metabolism. Notably, RBC and plasma data converged on the enrichment of vitamin B6 metabolism, suggesting a shared metabolic adap tation that may play a critical role in managing oxidative stress and supporting amino acid metabolism under reduced G6PD capacity (Supplemental Figure S3A,B).

Given the role of energy metabolism in ion homeostasis, which in turn impacts RBC morphology and deformability [67,68,69], we sought to determine whether these metabolic changes were accompanied by differences in trace metals. RBC profiling was conducted through inductively coupled plasma mass spectrometry (ICP-MS) quantified exercise-induced changes in intracellular metals. Several metals, such as manganese and cobalt, were elevated upon exercise but not different on the basis of G6PD status. In contrast, we observed post-exercise increases in intracellular calcium in hG6PD_ND_ but not hG6PD_A−_ mouse RBCs, while magnesium was significantly decreased post-exercise in the hG6PD_A−_ RBCs (Figure 2E and Supplemental Table S1). Magnesium is important for RBC membrane integrity, although through SEM analyses, no significant differences were found between hG6PD_ND_ and hG6PD_A−_ RBCs (Figure 2F and details elaborated in Supplemental Figure S2D). Exercise was accompanied by elevation in intracellular sodium and decreases in RBC potassium in hG6PD_ND_ mouse RBCs, while hG6PD_A−_ RBCs showed pre-exercise levels comparable to post-exercise in non-deficient mice, with no significant additional effect of exercise (Supplemental Figure S2C), consistent with a stress phenotype at baseline. Copper, zinc, and selenium all increased after exercise in non-deficient mice (albeit not significantly), with no significant changes in hG6PD_A−_ (Supplemental Figure S2C).

### 3.3. Proteome-Constrained Modeling Highlights hG6PD_A−_ RBCs Reprogram Metabolism to Enhance Oxygen Delivery

**Figure 3:**
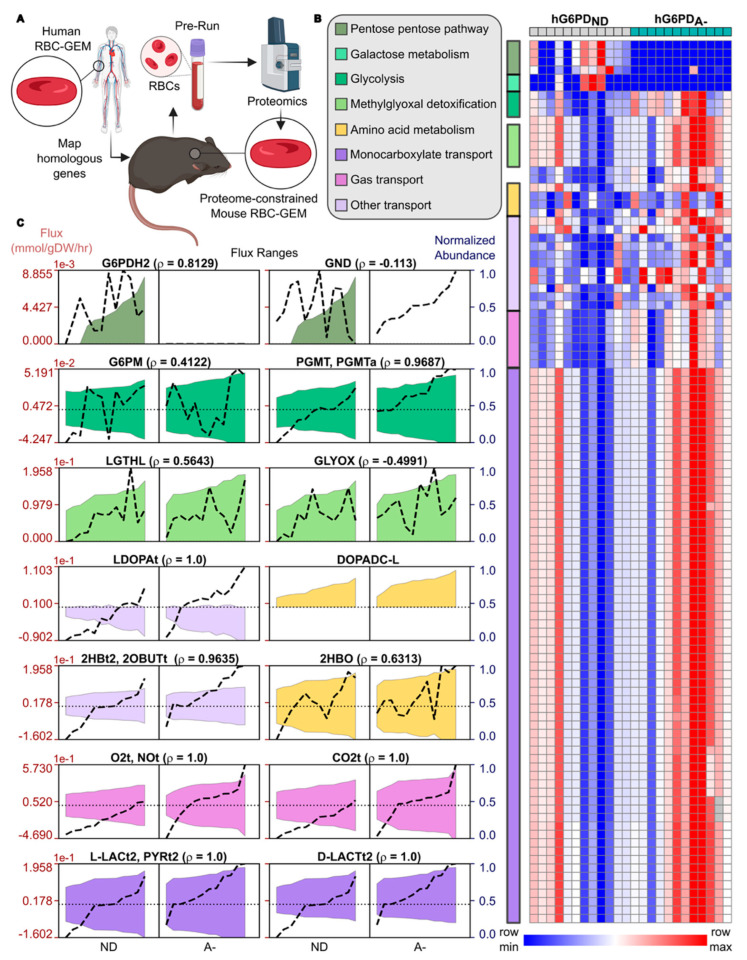
Proteome-constrained modeling of mice RBCs pre-exercise. (**A**) Creation of proteome-constrained models for mice RBCs from a human RBC genome-scale metabolic reconstruction (RBC-GEM) and proteomic data from mice, pre-exercise. (**B**) Reaction flux ranges considered statistically significant via Mann–Whitney U tests (*p* < 0.05) across humanized mice expressing G6PD canonical non-deficient protein (ND) or the African (A−) variant. (**C**) Statistically significant flux ranges compared across samples. Min–max normalized enzyme abundances are represented with the dashed line. Reaction and heatmap data can be found in the Supplemental Materials.

To understand the capacity of the RBC proteome for metabolic reprogramming, we formulated proteome-constrained models of mice RBCs from hG6PD_ND_ and hG6PD_A−_ pre-exercise proteomic data (Figure 3A). Using proteome-constrained flux variability analysis, we computed the effective flux ranges corresponding to the proteome. Analyses of statistically significant fluxes highlighted alterations in flux capacity in carbohydrate metabolism and membrane transport due to differences in protein levels with respect to G6PD status (Figure 3B and Supplemental Table S2). Predicted flux through the PPP decreased significantly for hG6PD_A−_ mice (Figure 3C). However, reaction flux increased for mutarotation of glucose 6-phosphate (G6PM) and for isomerization between glucose 1-phosphate and glucose 6-phosphate, consistent with predicted dependence of the phosphoglucomutase reaction on enzyme abundance (PGMT and PGMTa, ρ = 0.9687). Furthermore, models predicted an increased flux capacity in hG6PD_A−_ mice for detoxification of methylglyoxal, a glycolytic byproduct, through the glyoxalase pathway (LGTHL and GLYOX) to form D-lactate. The RBCs of hG6PD_A−_ mice also elucidated an increased capacity to exchange monocarboxylates such as pyruvate, L-lactate, and D-lactate with plasma due to increased abundance of monocarboxylate transporter (L-LACt2, PYRt2, and D-LACt2, ρ = 1). The increased capacity to exchange monocarboxylates 2-hydroxybutyrate and 2-ketobutyrate with plasma (2HBt2 and 2OBUTt, ρ = 0.9635) may facilitate increased oxidoreductase activity via L-lactate dehydrogenase within RBCs (2HBO). RBCs of hG6PD_A−_ were also predicted to have increased uptake capacity for L-dopa (LDOPAt, ρ = 1), which can undergo decarboxylation to L-dopamine (DOPADC-L) via methemoglobin and other ferriheme proteins [70]. Notably, proteome-constrained modeling predicted an increased capacity to exchange oxygen, nitric oxide, and carbon dioxide due to increased abundance of channel proteins (O2t, NOt, and CO2t, ρ = 1), thus providing additional support to our hypothesis that metabolic reprogramming in RBCs enhances exercise tolerance in hG6PD_A−_ mice by increasing oxygen delivery to fuel mitochondria of other tissues.

### 3.4. Skeletal Muscle Omics Profiles Reveal Higher Levels of Oxidation and Cellular Regulation

**Figure 4:**
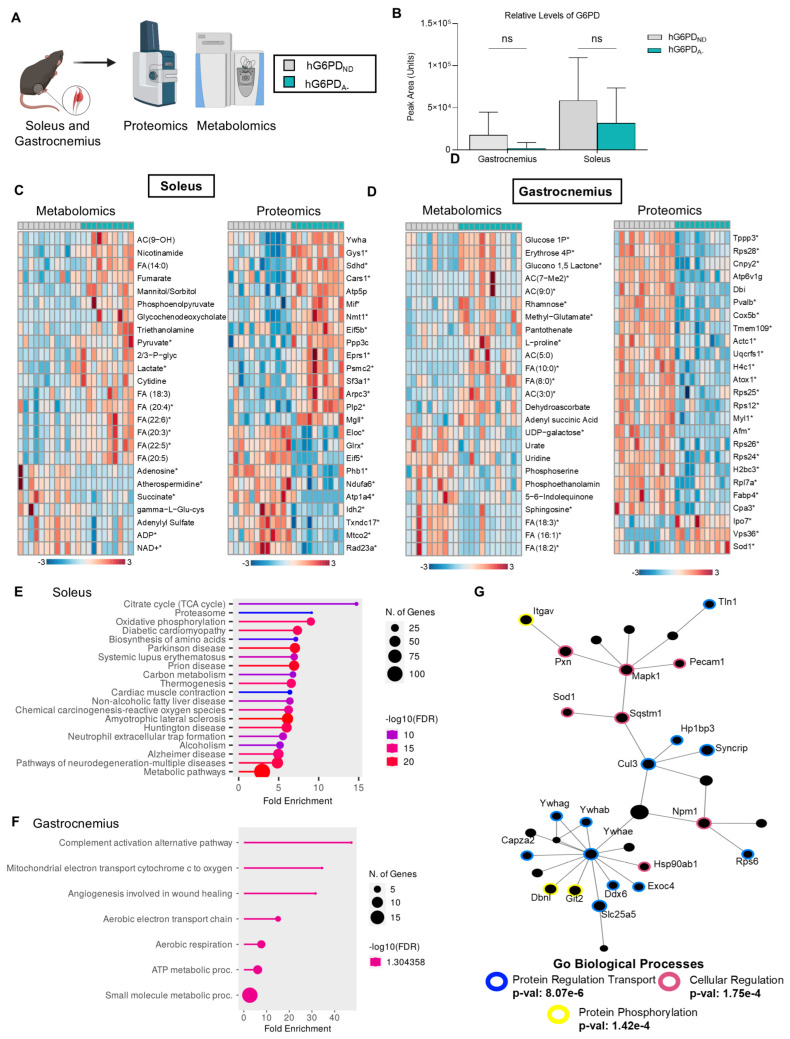
Proteomics and metabolomics analysis of muscles revealed both tissue- and genotype-specific differences. (**A**) Muscle tissues (gastrocnemius and soleus) were collected from hG6PD_ND_ and hG6PD_A−_ mice post-exercise. (**B**) Relative levels of G6PD show significant protein expression between the muscles organs. (**C**) Soleus and (**D**) gastrocnemius show significant genotype-specific differences for each tissue type, as measured by ANOVA. Protein abundances were grouped as being significantly increased or significantly decreased, with significant analytes indicated with a *. (**E**) Pathway enrichment analysis of significant metabolites and proteins within the soleus. (**F**) Pathway enrichment analysis of significant metabolites and proteins within the gastrocnemius. (**G**) Network and GO analysis of significant proteins in hG6PD_A−_ muscle tissues. Significance: *: *p* < 0.05.

Without significant differences in cardiac function between the two models (Supplemental Figure S1A) and only minor metabolic adaptations in RBCs from hG6PD_A−_ mice, we sought alternative explanations for the increased exercise tolerance of the G6PDd mice. Therefore, we expanded our analysis to skeletal muscles, focusing on both fast-twitch (gastrocnemius) and slow-twitch (soleus) fibers (Figure 4A). G6PD expression levels were not significantly different when comparing hG6PD_ND_ and hG6PD_A−_ in each of the two muscle types (Figure 4B), consistent with an increased de novo synthesis of this protein in transcriptionally/translationally competent tissues like muscle (unlike anucleated RBCs), to compensate for the lower protein stability [71]. However, genotype-specific differences between the soleus (Figure 4C) and gastrocnemius (Figure 4D) revealed distinct molecular profiles between hG6PD_ND_ and hG6PD_A−_. The soleus, rich in mitochondria and fatigue-resistant Type I fibers, supports endurance activity. hG6PD_A−_ mice exhibited elevated late glycolytic metabolites (phosphoglycerate, phosphoenolpyruvate, pyruvate, lactate), fumarate and polyunsaturated fatty acids (FA 18:3, 20:3, 20:4, 20:5, 22:5, 22:6). Proteomics revealed upregulation of succinate dehydrogenase (SDHD), linking increased fumarate levels to enhanced TCA cycle flux. These findings suggest upregulated glycolysis and fatty acid metabolism in hG6PD_A−_ mice, aligning with improved exercise tolerance, though steady-state data limit flux interpretation. Pathway analysis confirmed increased glycolysis, fatty acid metabolism, oxidative phosphorylation, and amino acid biosynthesis in the soleus (Figure 4E). Interestingly we also see the gastrocnemius increased within ATP production and aerobic respiration (this is due to the muscle being Type IIa and Type IIx) (Figure 4F). Gene ontology (GO) analysis of the top upregulated proteins in hG6PD_A−_ highlighted enrichment in pathways related to protein transport, phosphorylation, and cellular energy metabolism (Figure 4D and Supplemental Table S2).

### 3.5. Impact of G6PD Status and Exercise on Spleen, Kidney, and Liver

**Figure 5:**
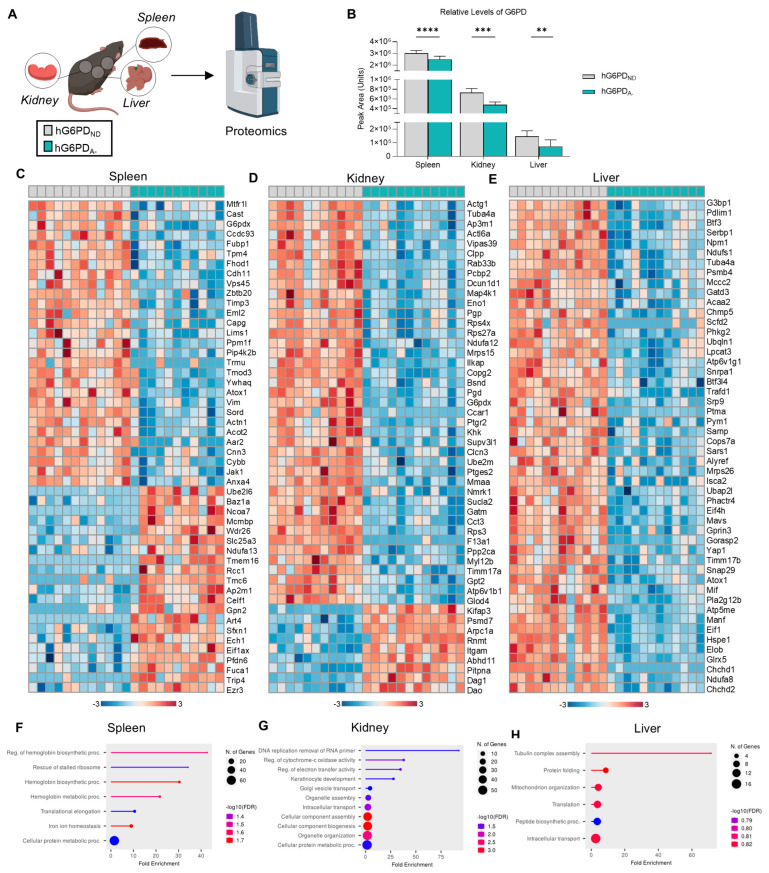
Proteomics analysis of tissue revealed both tissue- and genotype-specific differences. (**A**) Muscle tissues (spleen, liver, and kidney) were isolated from hG6PD_ND_ and hG6PD_A−_ mice for proteomics analysis. (**B**) Relative levels of G6PD show significant decreases in these three organs. (**C**–**E**) Significant genotype specific differences were observed for each tissue type, as measured by ANOVA. Protein abundances were grouped as being significantly increased or significantly decreased. (**F**–**H**) Pathway enrichment analysis of significant proteins. Significance: **: *p* < 0.01, ***: *p* < 0.001, ****: *p* < 0.0001.

G6PDd RBCs are susceptible to increased lysis in circulation when under oxidative stress. However, no differences in plasma Hb levels were observed between the G6PD_ND_ and G6PD_A−_ mice after exercise. We next questioned whether exercise promoted extravascular hemolysis of RBCs through sequestration and erythrophagocytosis by splenic and liver macrophages. As such, we next examined key organs involved in RBC turnover or erythrophagocytosis: spleen, liver, or kidney, respectively (Figure 5A). Significant differences in G6PD expression were observed between hG6PD_ND_ and hG6PD_A−_ in each of these tissues, with lower levels in deficient mice compared to controls (Figure 5B and details elaborated in Supplemental Figure S2E). Heatmaps of the top 50 differentially expressed proteins highlighted clear distinctions between the genotypes across all three organs (Figure 5C–E). Using significantly altered proteins in both hG6PD_ND_ and hG6PD_A−_ models for GO analysis, we identified notable enrichment in pathways related to hemoglobin and iron homeostasis (Figure 5F). This highlights potential disruptions in oxygen transport and iron regulation, key components of systemic metabolic balance. In the kidney, we observed enrichment for electron transport activity and cytochrome c oxidase activity, suggesting an increase in oxidative phosphorylation and heightened mitochondrial activity to meet energy demands under stress (Figure 5G). Meanwhile, pathway enrichment analysis in the liver highlighted increases in protein folding and translation processes, which may reflect adaptive responses to maintain cellular integrity and biosynthetic capacity during metabolic stress (Figure 5H). Together, these findings underscore tissue-specific metabolic adaptations and emphasize the interplay between systemic and organ-level responses in these models.

**Figure 6:**
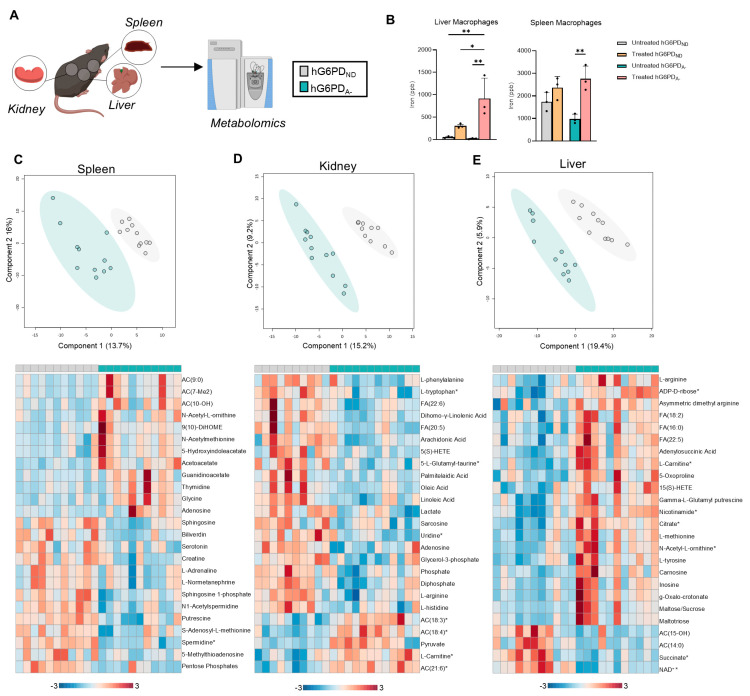
Metabolomics analysis of tissue revealed both tissue- and genotype-specific differences. (**A**) Tissues were collected from hG6PD_ND_ and hG6PD_A−_ mice. (**B**) Liver and spleen iron levels for macrophages through erythrophagocytosis. Treated = with RBCs and Untreated = without RBCs. Principal component analysis and heatmaps between hG6PD_ND_ and hG6PD_A−_ genotypes showed significant differences in (**C**) spleen, (**D**) kidney, and (**E**) liver, as measured by ANOVA with significantly different metabolites indicated with a *. Significance: *: *p* < 0.05, **: *p* < 0.01.

To further investigate the metabolic changes underlying cellular processes, particularly in iron homeostasis and cellular metabolism, we conducted high-throughput metabolomics on the spleen, kidney, and liver (Figure 6A). In parallel, ICP-MS analysis of tissue extracts was performed to quantify macrophage iron levels, hypothesized to increase due to enhanced erythrophagocytosis by resident macrophages. These analyses revealed a significant increase in iron levels, corroborating elevated erythrophagocytosis in both the liver and spleen of hG6PD_A−_ mice compared to non-deficient controls (Figure 6B). Principal component analysis (PCA) demonstrated clear separation between the metabolic profiles of hG6PD_ND_ and hG6PD_A−_ genotypes, underscoring the distinct metabolic reprogramming driven by G6PDd (Figure 6C–E, heat maps represent top 50 metabolites by *p* value). In the spleen, hG6PD_A−_ mice exhibited increased levels of ACs alongside decreased levels of polyamine metabolites and biliverdin, suggesting significant shifts in lipid metabolism and heme catabolism (Figure 6C). Enrichment analysis of the significantly altered metabolites in the spleen of hG6PD_A−_ mice highlighted arginine biosynthesis, amino acid metabolism, and, notably, riboflavin (B_2_) metabolism, underscoring potential changes in energy production and oxidative stress pathways (Supplemental Figure S3C). The kidney, like the spleen, exhibited increases in ACs and shifts in key metabolic pathways (Figure 6D). Enrichment analysis revealed pathways associated with mitochondrial metabolism, including the TCA cycle, pyruvate metabolism, and glycolysis, suggesting metabolic adaptation (Supplemental Figure S3D). The liver displayed pronounced increases in FAs, ACs, and TCA cycle intermediates, reflecting enhanced metabolic activity and mitochondrial function in response to G6PD deficiency (Figure 6E). Enrichment analysis further emphasized these pathways, with significant increases in amino acid metabolism and fatty acid biosynthesis, suggesting heightened biosynthetic and energy-generating activity (Supplemental Figure S3E). Collectively, these findings highlight tissue-specific metabolic adaptations to G6PDd, with the liver and spleen playing central roles in managing systemic impacts of erythrophagocytosis and altered iron metabolism. The kidney, while less affected, appears to support metabolic homeostasis through mitochondrial activity in a more limited capacity.

## 4. Discussion

Our study elucidates metabolic adaptations in hG6PD_A−_ mice that enhance exercise capacity despite reduced enzymatic activity of the rate-limiting step of the PPP. Notably, hG6PD_A−_ mice demonstrated a 10.8% faster CS compared to controls, suggesting metabolic compensation. Further investigations characterized changes in RBC metabolism, including increased synthesis of 2,3-BPG in these mice, consistent with the expected increase in glycolytic fluxes, which in turn fuel the Rapoport–Luebering shunt, upon the genetic ablation of the PPP. Despite this metabolic shift, there was no evidence of substantial oxidative stress within RBCs, suggesting that red cell adaptation may be anticipatory or compensatory rather than reactive to oxidant damage. Instead, our data point to skeletal muscle as the primary site of exercise-induced stress in hG6PD_A−_ mice. This finding contrasts with our prior observations of hG6PD_Med−_ mice where increased CS was noted, but the more severe enzymatic deficiency was compensated for with cardiovascular remodeling rather than altered RBC metabolism. These distinct adaptive responses highlight the differential impact of the severity of G6PDd on systemic physiology and performance as a function of the spectrum of severity of the genetically linked enzymopathy.

**Figure 7:**
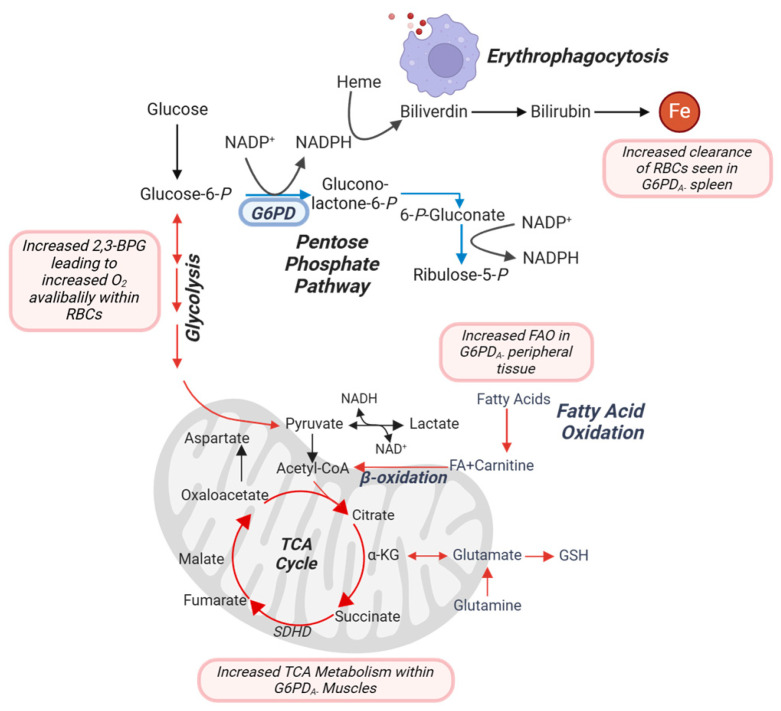
Metabolic overview of key pathways highlighted in this study. Figure created with BioRender.com.

Proteomic and metabolomic analyses across skeletal muscle revealed distinct, tissue-specific metabolic adaptations in hG6PD_A−_ mice driven by the energy demands of exercise. In slow-twitch fibers (soleus), elevated oxidative phosphorylation and TCA cycle activity were associated with the upregulation of SDHD, a key subunit of complex II in the mitochondrial electron transport chain [72]. This highlights increased mitochondrial efficiency and energy production, enabling endurance-related performance. Conversely, fast-twitch fibers (gastrocnemius) showed increased reliance on glycolysis and ATP production, reflecting a shift toward anaerobic energy generation suited for short bursts of activity (Figure 7). These differences underscore how mitochondrial metabolism is tailored to muscle fiber type in response to G6PDd and exercise stress [73]. Together, these findings support the idea that, in hG6PD_A−_ mice, skeletal muscle is the primary site of metabolic adaptation to exercise, while RBCs, though metabolically responsive, do not appear to experience significant oxidative stress.

Peripheral tissues (liver, spleen, and kidney) exhibited distinct reprogramming to manage systemic metabolic demands. The liver emerged as a critical hub for systemic energy regulation, with elevated levels of FAs, ACs, and TCA cycle intermediates. These findings suggest that the liver compensates for G6PDd by enhancing biosynthetic and oxidative stress mitigation pathways, supporting RBC turnover and maintaining systemic homeostasis [74,75]. Most notably, the spleen demonstrated increased erythrophagocytosis, as evidenced by elevated iron levels and shifts in lipid metabolism and heme catabolism (Figure 7). This reflects its role in managing RBC turnover and oxidative stress. The kidney, while showing modest metabolic changes, displayed enrichment in mitochondrial pathways such as the TCA cycle and pyruvate metabolism [76], indicating a supporting role in maintaining energy balance under stress. Together, these findings reveal that hG6PD_A−_ mice rely on systemic metabolic plasticity rather than localized adaptations. Enhanced mitochondrial function in the soleus, coupled with liver-driven metabolic regulation and spleen-mediated RBC turnover, collectively support the increased exercise tolerance observed in deficient mice [77,78]. These systemic adaptations highlight the interplay between tissue-specific energy production and oxidative stress management in overcoming the challenges of G6PDd.

In our previously published hG6PD_Med−_ model, cardiac adaptations were the primary drivers of improved exercise performance. Enhanced stroke volume, increased cardiac output, and mitochondrial biogenesis within cardiac and skeletal muscle tissues underpinned the observed 8% improvement in CS [39]. This was accompanied by changes in sarcomere length and composition, as well as elevated protein turnover and mitophagy. In contrast, hG6PD_A−_ mice exhibited no significant baseline cardiovascular differences, and instead relied on metabolic reprogramming in RBCs and other tissues, including liver, spleen, and kidney, to sustain higher exercise capacity. Elevated post-exercise RBCs levels of 2,3-BPG facilitated oxygen offloading, optimizing oxygen delivery in the setting of exercise-induced hypoxia. Additionally, increased reliance on glycolysis and fatty acid oxidation accentuates a shift toward enhanced mitochondrial energy production in plasma (see model in Figure 7). Interestingly, while both G6PDd models demonstrated heightened mitochondrial activity, their mechanisms differed. hG6PD_Med−_ mice exhibited systemic mitochondrial adaptations with marked cardiovascular remodeling, while hG6PD_A−_ mice emphasized metabolic plasticity within RBCs and peripheral tissues, including the liver, spleen, and kidney. Proteomic analyses revealed enrichment of pathways related to energy metabolism and protein transport, further supporting the notion that RBC metabolism plays a pivotal role in compensating for the enzymatic deficiency in the hG6PD_A−_ model.

Our findings underscore the utility of exercise as a physiologically relevant and minimally invasive approach to induce acute oxidative stress in vivo, offering a powerful tool for studying both short- and long-term molecular responses. By leveraging this model, we can generate hypotheses regarding the broader implications of oxidative stress in G6PD-deficient individuals. The metabolic flexibility observed in hG6PD_A−_ mice reveals unique adaptations that may optimize energy utilization and mitigate oxidative damage under controlled conditions. These insights challenge the traditional notion that strenuous exercise is universally detrimental for G6PD-deficient individuals, instead suggesting a context-dependent benefit that warrants further exploration [79]. Our findings in hG6PD_A−_ mice challenge assumptions that RBCs are a major target of exercise-induced oxidative injury in mild G6PDd. Instead, the burden of adaptation appears to be managed primarily by skeletal muscle and peripheral organs, emphasizing a tissue-specific stress response.

However, the absence of significant cardiovascular remodeling in the hG6PD_A−_ model indicates that these advantages may vary based on the severity of the deficiency and other physiological factors. Moving forward, our findings highlight the need to dissect the molecular pathways underlying these adaptations, both in animal models and human studies. Such investigations have the potential to inform targeted interventions aimed at enhancing metabolic resilience and mitigating risks associated with oxidative stress in G6PDd. Future studies should focus on the long-term implications of these metabolic and cardiovascular adaptations, particularly in the context of aging and the increased risk of chronic conditions such as cardiovascular disease [36,80]. While young G6PDd mice exhibit remarkable resilience and compensatory mechanisms, the cumulative effects of oxidative stress throughout a lifetime may diminish these advantages [81], resulting in accelerated aging [82]. Age-associated declines in metabolic and cardiovascular function could exacerbate vulnerabilities, particularly in conditions such as pulmonary arterial hypertension and atherosclerosis, which are more prevalent in older individuals with G6PDd [32,36,83]. Understanding these progressive changes will not only provide insights into the lifespan of the observed adaptations but may also inform therapeutic strategies aimed at mitigating long-term health risks in this population [31,84].

In conclusion, our study underscores the remarkable metabolic plasticity of hG6PD_A−_ mice, enabling them to not only compensate for enzymatic deficiencies but also enhance exercise performance. By contrasting these findings with the cardiovascular adaptations observed in hG6PD_Med−_ mice, we provide novel insights into the diverse physiological responses to exercise in G6PDd as a function of the spectrum of variation in G6PD enzyme deficiencies. These results highlight the need for personalized exercise recommendations and therapeutic strategies that account for the specific metabolic and cardiovascular profiles of G6PDd individuals as a function of the degree of deficiency, ultimately aiming to improve their overall health and well-being. As G6PD expression and activity vary with sex (X-linked) and decline with age, even in healthy (G6PD sufficient) individuals, some of the findings reported herein may inform the interpretation of age-related physiological responses to exercise [14,85].

## Data Availability

The original contributions presented in this study are included in the Supplementary Material. Further inquiries can be directed to the corresponding author.

## Supplementary Materials

The following supporting information can be downloaded at: https://www.mdpi.com/article/10.3390/antiox14080927/s1, Supplemental Table S1 contains metabolomics, ICP, and critical speed raw data used in this paper. Supplemental Table S2 contains all raw proteomics data used within this paper. Supplemental Material PDF final contains supporting figures.
