# Supplementary material for: Increased Exercise Tolerance in G6PD African Variant Mice Driven by Metabolic Adaptations and Erythrophagocytosis
Source: Antioxidants (Basel). 2025 Jul 29;14(8):927. doi: 10.3390/antiox14080927 (PMC12382757; doi:10.3390/antiox14080927)
Supplement: Supplementary file 1 [file antioxidants-14-00927-s001.zip › 3712506_Supplementary Material_Final.pdf]

SUPPLEMENTARY MATERIAL

Increased Exercise Tolerance in G6PD African Variant Mice Driven by Metabolic Adaptations and Erythrophagocytosis

Francesca I Cendali,[1] Abby L. Grier,[1] Christina Lisk,[2] Monika Dzieciatkowska,[1] Zachary Haiman, [1] Julie A Reisz,[1] Julie Harral,[3] Daniel Stephenson,[1] Ariel M Hay,[6] Eric P. Wartchow,[4] Paul W. Buehler,[7,8] Kirk C. Hansen,[1] Travis Nemkov,[1] James C. Zimring,[6] David C. Irwin,[2] Angelo D'Alessandro[1,*]

[1] Department of Biochemistry and Molecular Genetics, University of Colorado, Anschutz Medical Campus, Aurora, Colorado 80045, USA

[2] Cardiovascular and Pulmonary Research Laboratory, Department of Medicine, University of Colorado, Anschutz Medical Campus, Aurora, Colorado 80045, USA

[3] Department of Medicine, University of Colorado Denver, Aurora, Colorado, 80045 USA

[4] Department of Pathology, Children's Hospital Colorado, Aurora, Colorado 80045, USA

[5] Electron Microscopy Core Facility, University of Colorado, Anschutz Medical Campus, Aurora, Colorado 80045, USA

[6] Department of Pathology, University of Virginia School of Medicine, Charlottesville, Virginia 22903, USA

[7] The Center for Blood Oxygen Transport, Department of Pediatrics, University of Maryland School of Medicine, Baltimore, Maryland 21201, USA

***Corresponding author:**

Angelo D'Alessandro, PhD
Department of Biochemistry and Molecular Genetics
University of Colorado Anschutz Medical Campus
12801 East 17th Ave., Aurora, CO 80045
Phone +1 303-724-0096
E-mail: angelo.dalessandro@cuanschutz.edu
www.dalessandrolab.com
@dalessandrolab

Running title: *Increased exercise tolerance in G6PD African deficient mice*

TABLE OF CONTENTS

SUPPLEMENTARY FIGURES **2**

SUPPLEMENTARY FIGURE 1 2
SUPPLEMENTARY FIGURE 2 3
SUPPLEMENTARY FIGURE 3 4

SUPPLEMENTARY METHODS 6

SUPPLEMENTARY TABLE 1 .... **XLS**

SUPPLEMENTARY TABLE 2 .... **XLS**

Characteristic	Median $G6PD_{ND}$ (n=12)	Median $G6PD_{A-}$ (n=12)	Fold Change	Significance
Final Body Weight (g)	28.05 ± 1.71 (n=12)	28.00 ± 1.67 (n=12)	1.00	n.s.
Systemic Systolic Blood Pressure (mmHg)	52.72 ± 9.19 (n=7)	50.87 ± 10.89 (n=4)	0.96	n.s.
Systemic Diastolic Blood Pressure (mmHg)	20.19 ± 6.46 (n=7)	23.11 ± 6.01 (n=4)	1.14	n.s.
Systemic Mean Blood Pressure (mmHg)	31.91 ± 7.04 (n=7)	33.43 ± 6.47 (n=4)	1.05	n.s.
Systemic Pulse Pressure (mmHg)	35.16 ± 5.36 (n=7)	29.72 ± 9.90 (n=4)	0.85	n.s.
Heart Rate (bpm)	569.50 ± 30.20 (n=12)	555.00 ± 2.06 (n=12)	0.97	n.s.
Pulmonary Artery Systolic Blood Pressure (mmHg)	25.11 ± 0.83 (n=4)	24.99 ± 2.06 (n=10)	1.00	n.s.
Pulmonary Artery Diastolic Blood Pressure (mmHg)	8.88 ± 3.12 (n=4)	12.13 ± 2.12 (n=10)	1.37	n.s.
Pulmonary Artery Mean Blood Pressure (mmHg)	14.33 ± 1.89 (n=4)	16.42 ± 1.96 (n=10)	1.15	n.s.
Pulmonary Artery Pulse Pressure (mmHg)	16.33 ± 3.76 (n=4)	12.49 ± 1.86 (n=10)	0.76	n.s.
Cardiac Output (mL/min)	7.93 ± 3.33 (n=8)	15.76 ± 4.34 (n=3)	1.99	*
Cardiac Index (mL/min·g)	0.30 ± 0.11 (n=8)	0.58 ± 0.17 (n=3)	1.93	*
Pulmonary Vascular Resistance (mmHg·min/mL)	1.42 ± 1.26 (n=4)	1.38 ± 1.13 (n=10)	0.97	n.s.
Systemic Vascular Resistance (mmHg·min/mL)	3.61 ± 2.16 (n=6)	2.29 ± 0.71 (n=3)	0.63	n.s.
Pulmonary Arterial Compliance (mL/mmHg)	1.55 ± 0.74 (n=3)	1.58 ± 0.59 (n=10)	1.02	n.s.

Supplementary Figure S1. Hemodynamics Results. Cardiac and morphological data for mice with the G6PD human canonical and African variant, collected at take down during hemodynamics. Fold changes were calculated using the medians for each group, and p-values were generated for each comparison using Student's t-test. * ($p \leq 0.05$) was used to label significance.

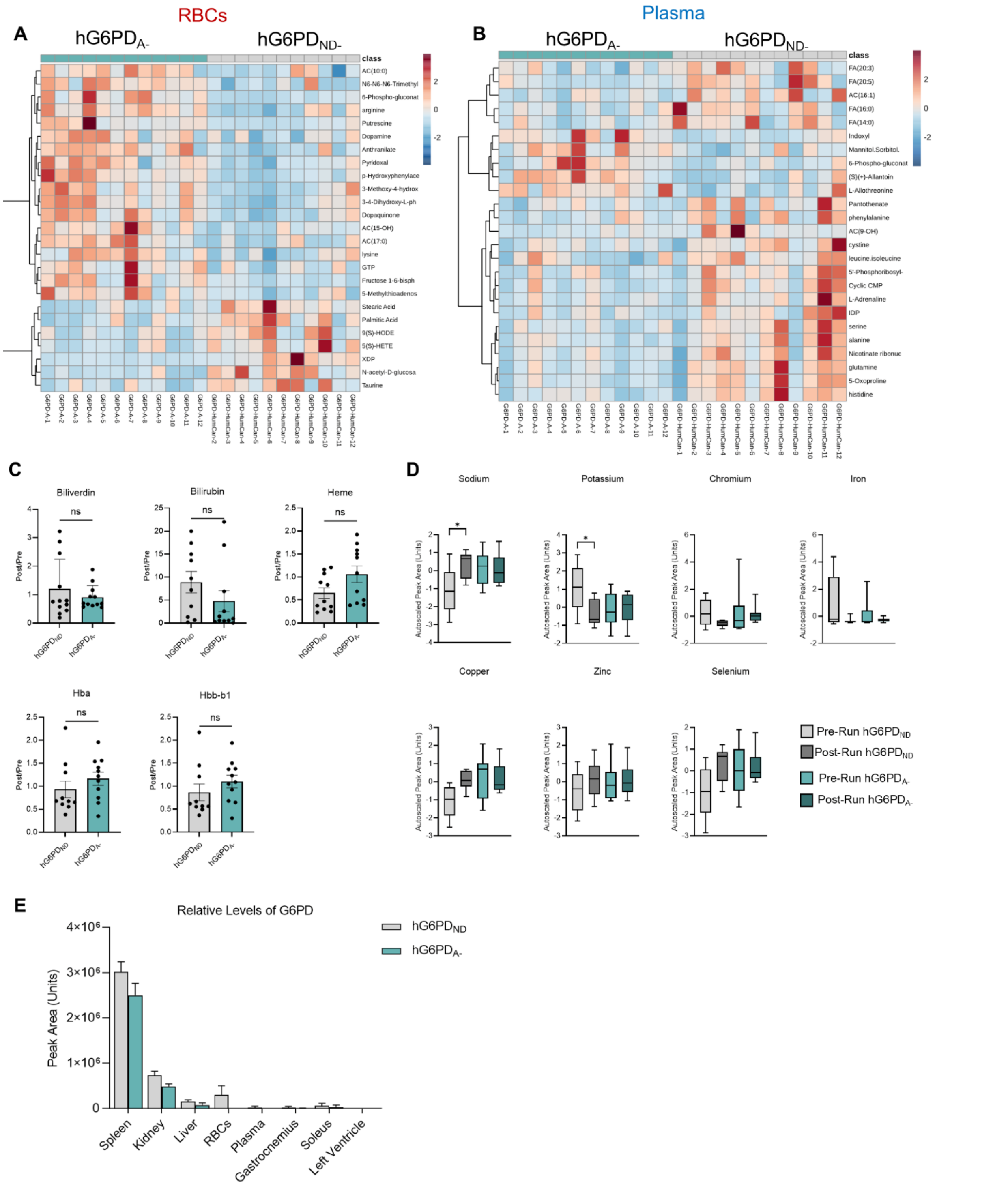

Supplemental Figure S2. Pre-Post Blood Analysis. A) RBC and B) Plasma Heat map of top 25 metabolites significantly different between hG6PD$_{ND}$ and hG6PD$_{A-}$ taken from the post/pre fold change, as measured by ANOVA. Auto scaled metabolite abundances were compared pre and post exercise between hG6PD$_{ND}$ and hG6PD$_{A-}$ genotypes C.) Marker for hemolysis D) ICP RBC pre-post blood changes E) Total G6PD expression in all tissues analyses. (Significance *: $p < 0.05$, **: $p < 0.01$, ****: $p < 0.0001$)

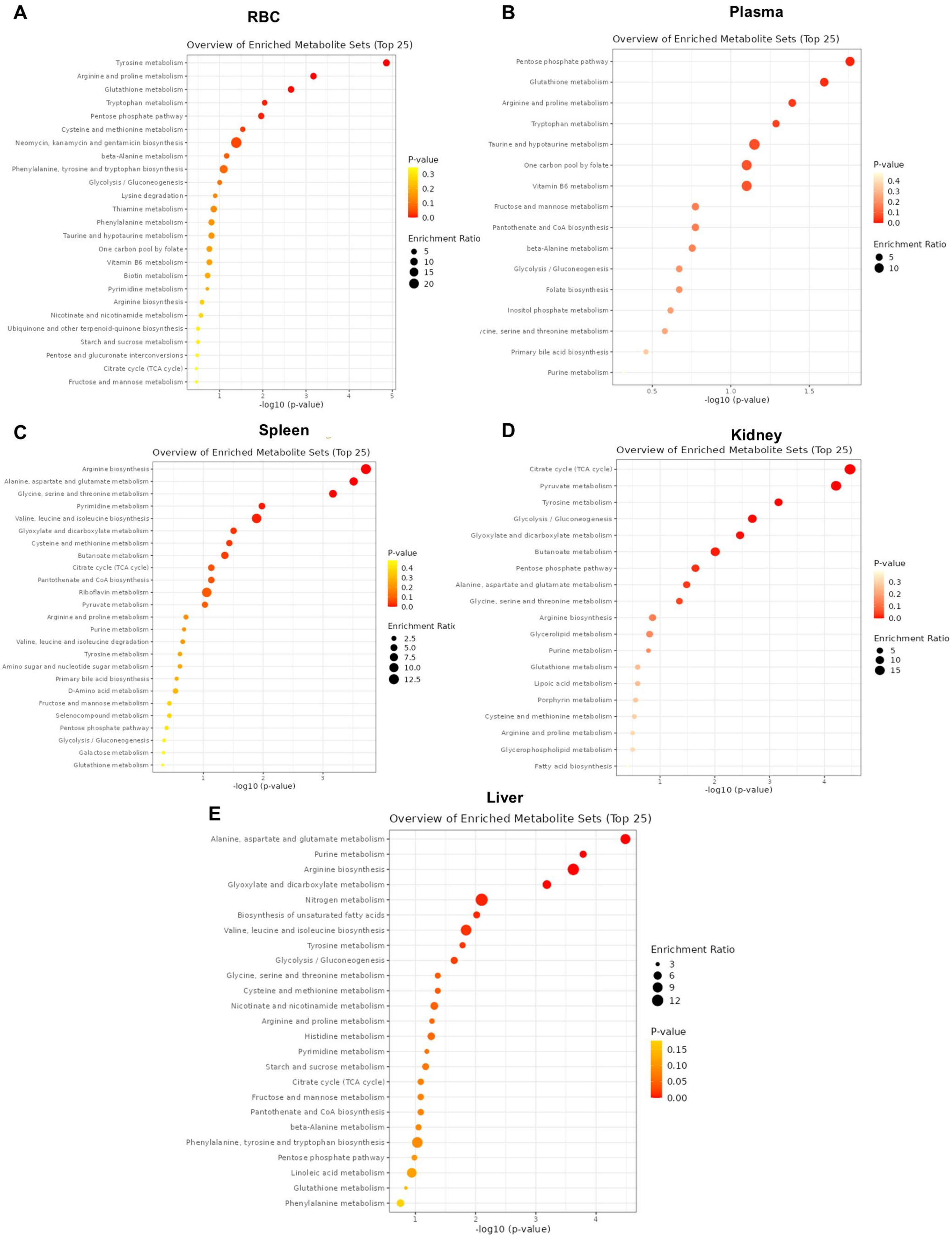

A
RBC
Overview of Enriched Metabolite Sets (Top 25)
Tyrosine metabolism
Arginine and proline metabolism
Glutathione metabolism
Tryptophan metabolism
Pentose phosphate pathway
Cysteine and methionine metabolism
Neomycin, kanamycin and gentamicin biosynthesis
beta-Alanine metabolism
Phenylalanine, tyrosine and tryptophan biosynthesis
Glycolysis / Gluconeogenesis
Lysine degradation
Thiamine metabolism
Phenylalanine metabolism
Taurine and hypotaurine metabolism
One carbon pool by folate
Vitamin B6 metabolism
Biotin metabolism
Pyrimidine metabolism
Arginine biosynthesis
Nicotinate and nicotinamide metabolism
Ubiquinone and other terpenoid-quinone biosynthesis
Starch and sucrose metabolism
Pentose and glucuronate interconversions
Citrate cycle (TCA cycle)
Fructose and mannose metabolism
-log10 (p-value)
P-value
Enrichment Ratio
B
Plasma
Overview of Enriched Metabolite Sets (Top 25)
Pentose phosphate pathway
Glutathione metabolism
Arginine and proline metabolism
Tryptophan metabolism
Taurine and hypotaurine metabolism
One carbon pool by folate
Vitamin B6 metabolism
Fructose and mannose metabolism
Pantothenate and CoA biosynthesis
beta-Alanine metabolism
Glycolysis / Gluconeogenesis
Folate biosynthesis
Inositol phosphate metabolism
/cine, serine and threonine metabolism
Primary bile acid biosynthesis
Purine metabolism
-log10 (p-value)
P-value
Enrichment Ratio
C
Spleen
Overview of Enriched Metabolite Sets (Top 25)
Arginine biosynthesis
Alanine, aspartate and glutamate metabolism
Glycine, serine and threonine metabolism
Pyrimidine metabolism
Valine, leucine and isoleucine biosynthesis
Glyoxylate and dicarboxylate metabolism
Cysteine and methionine metabolism
Butanoate metabolism
Citrate cycle (TCA cycle)
Pantothenate and CoA biosynthesis
Riboflavin metabolism
Pyruvate metabolism
Arginine and proline metabolism
Purine metabolism
Valine, leucine and isoleucine degradation
Tyrosine metabolism
Amino sugar and nucleotide sugar metabolism
Primary bile acid biosynthesis
D-Amino acid metabolism
Fructose and mannose metabolism
Selenocompound metabolism
Pentose phosphate pathway
Glycolysis / Gluconeogenesis
Galactose metabolism
Glutathione metabolism
-log10 (p-value)
P-value
Enrichment Ratic
D
Kidney
Overview of Enriched Metabolite Sets (Top 25)
Citrate cycle (TCA cycle)
Pyruvate metabolism
Tyrosine metabolism
Glycolysis / Gluconeogenesis
Glyoxylate and dicarboxylate metabolism
Butanoate metabolism
Pentose phosphate pathway
Alanine, aspartate and glutamate metabolism
Glycine, serine and threonine metabolism
Arginine biosynthesis
Glycerolipid metabolism
Purine metabolism
Glutathione metabolism
Lipoic acid metabolism
Porphyrin metabolism
Cysteine and methionine metabolism
Arginine and proline metabolism
Glycerophospholipid metabolism
Fatty acid biosynthesis
-log10 (p-value)
P-value
Enrichment Ratio
E
Liver
Overview of Enriched Metabolite Sets (Top 25)
Alanine, aspartate and glutamate metabolism
Purine metabolism
Arginine biosynthesis
Glyoxylate and dicarboxylate metabolism
Nitrogen metabolism
Biosynthesis of unsaturated fatty acids
Valine, leucine and isoleucine biosynthesis
Tyrosine metabolism
Glycolysis / Gluconeogenesis
Glycine, serine and threonine metabolism
Cysteine and methionine metabolism
Nicotinate and nicotinamide metabolism
Arginine and proline metabolism
Histidine metabolism
Pyrimidine metabolism
Starch and sucrose metabolism
Citrate cycle (TCA cycle)
Fructose and mannose metabolism
Pantothenate and CoA biosynthesis
beta-Alanine metabolism
Phenylalanine, tyrosine and tryptophan biosynthesis
Pentose phosphate pathway
Linoleic acid metabolism
Glutathione metabolism
Phenylalanine metabolism
-log10 (p-value)
Enrichment Ratio
P-value

Supplementary Figure S3. Metabolomics Pathway enrichment analysis for A) RBC B.) Plasma C) Spleen D) Kidney E) Liver taken from the most significant metabolites present in $G6PD_{A-}$

Supplemental Methods

Treadmill exercise and constant speed tests

Prior to the determination of CS, mice completed a treadmill familiarization phase, which consisted of four ~5 min runs on a motor-driven rodent treadmill (Exer 3/6, Columbus Instruments, Columbus, Ohio, USA). For the first several runs, the treadmill speed was maintained at 10-15 m/min (up a 5% grade, which was maintained throughout all treadmill tests). For the last several runs, the speed of the treadmill increased progressively over the last minute to ~30-35 m/min to familiarize the mice with high-speed running. Animals were encouraged to run with intermittent bursts of compressed room air aimed at the hind limbs from directly above the animal (so as not to push the mouse up the treadmill). All treadmill testing protocols were designed and conducted by experienced staff and strictly followed the guidelines set by the American Physiological Society's resource book for the design of animal exercise protocols. The CS was determined using a modified version of the methodology used by Copp et al, 2010 for rats, as we previously described in mice, following the guidelines set forth by Poole et al. After completion of the treadmill familiarization period, each mouse performed 3-5 runs to exhaustion, in random order, at a constant speed that resulted in fatigue between 1 and 15 minutes (speeds ranging from 30-50 m/min). Each test was performed on separate days with a minimum of 24 hours between tests. For each constant-speed trial, mice were given a 2-minute warm-up period where they ran at 15-20 m/min followed by a 1-minute period of quiet resting. To start the test, the treadmill speed was increased rapidly over a 10 second period to the desired speed at which point a stopwatch was started. Testing was terminated and time to exhaustion was measured to the nearest tenth of a second whenever the mouse could no longer maintain pace with the treadmill despite obvious exertion of effort. A successful constant-speed test was determined if 1) the mouse could quickly adapt to the treadmill speed at the beginning of the test (e.g., did not waste energy), 2) a noticeable change in gait occurred preceding exhaustion (i.e., lowering of the hindlimbs and rising of the snout), and 3) the animal's righting reflex was markedly attenuated when placed on their back in a supine position (an unexhausted quadruped will typically attempt to right themselves within ~1 second).

Hemodynamics

After the CS test, mice underwent terminal open chest right ventricular (RV) and left ventricular (LV) function measurements with a 1.2F, FTE-1212B-4018 pressure volume catheter (Transonic Systems Inc., Ithaca, NY) inserted by direct cardiac puncture. Mice were induced through inhaled isoflurane (4-5%), and tracheal incision (~ 1 cm) was performed. Next, a tracheal tube was inserted and connected to an Anesthesia Workstation or Hallowell EMC Microvent and an anesthetic plain was maintained at 1.0-2.5% isoflurane in 100% oxygen. After which, a thoracotomy was performed exposing the heart, the pericardium was resected, and a small hole made at the base of the RV/LR with a 30g needle for insertion of the pressure-volume catheter. Steady state hemodynamics are collected with short pauses in ventilation (up to 10 seconds) or high frequency oscillatory ventilation to eliminate ventilator artifact from the pressure-volume recordings. Occlusions of the inferior vena cava were performed by applying pressure to the inferior vena cava (up to 10 seconds) through the abdominal opening. After pressure volume and hemodynamic measurements completed mice were humanely euthanized by exsanguination and cervical dislocation. Data was recorded continuously using LabScribe2 and analyzed offline.

Mass spectrometry-based proteomics

Frozen tissues (10 mg) were powdered in liquid nitrogen using a ceramic mortar and pestle and lyophilized. Weighed tissue (approximately 8 mg of each) was homogenized in freshly prepared high-salt buffer (50 mM Tris-HCl, 3 M NaCl, 25 mM EDTA, 0.25% w/v CHAPS, pH 7.5) containing 1x protease inhibitor (Halt Protease Inhibitor, Thermo Scientific) at a concentration of 10 mg/mL. Homogenization took place in a bead beater (Bullet Blender Storm 24, Next Advance, 1 mm glass beads) for 3 min at 4 $^{\circ}$C. Samples were then spun for 20 min 18,000 x g at 4 $^{\circ}$C, and the supernatant was removed and precipitated with chloroform/methanol. The protein pellets after chloroform/methanol precipitation were solubilized in 4% SDS in 100 mM triethylammonium bicarbonate (TEAB) pH 7.0 lysis buffer and digested using S-Trap 96-well plates (Protifi, Huntington, NY) following the manufacturer's procedure.

Plasma and RBCs samples were digested and analyzed using methods previously described.[43] An aliquot of each peptide sample was loaded onto individual Evotips for desalting and washing. Peptides were separated on a Pepsep column, (150 um inter diameter, 15 cm) packed with ReproSil

C18 1.9 um, 120A resin using pre-set 30 samples per day gradient on the Evosep One system (Evosep, Odense, Denmark). The Evosep system was coupled to the timsTOF Pro mass spectrometer (Bruker Daltonics, Bremen, Germany) via the nano-electrospray ion source (Captive Spray, Bruker Daltonics). The mass spectrometer was operated in PASEF mode. The ramp time was set to 100 ms and 10 PASEF MS/MS scans per topN acquisition cycle were acquired. MS and MS/MS spectra were recorded from *m/z* 100 to 1,700. The ion mobility was scanned from 0.7 to 1.50 Vs/cm^2. Precursors for data-dependent acquisition were isolated within ± 1 Th and fragmented with an ion mobility-dependent collision energy, which was linearly increased from 20 to 59 eV in positive mode. Low-abundance precursor ions with an intensity above a threshold of 500 counts but below a target value of 20,000 counts were repeatedly scheduled and otherwise dynamically excluded for 0.4 min. Raw data files conversion to peak lists in the MGF format, downstream identification, validation, filtering and quantification were managed using FragPipe version 13.0. MSFragger version 3.0 was used for database searches against a mouse database with decoys and common contaminants added. The identification settings were as follows: Trypsin, Specific, with a maximum of 2 missed cleavages, up to 2 isotope errors in precursor selection allowed for, 10.0 ppm as MS1 and 20.0 ppm as MS2 tolerances; fixed modifications: Carbamidomethylation of C (+57.021464 Da), variable modifications: Oxidation of M (+15.994915 Da),), Hydroxylation of P (+15.994915 Da), Acetylation of protein N-term (+42.010565 Da), Pyrolidone from peptide N-term Q or C (-17.026549 Da), diglycine modified lysine - KGG (+114.0429 Da).

Mass spectrometry-based Metabolomics and Lipidomics

Metabolites from blood were extracted from 10 uL (1:10) and tissue specimens were powdered with a mortar and pestle in a liquid nitrogen bath, then extracted at 15 mg/mL with cold MeOH:MeCN:H2O (5:3:2, v:v:v). Suspensions were vortexed vigorously for 30 min at 4°C. Insoluble material was pelleted by centrifugation (18,213 *g*, 10 min, 4°C) and supernatants were isolated for analysis on a Thermo Vanquish UHPLC coupled to a Thermo Q Exactive MS as previously described in detail. Oxylipin analysis employed a Vanquish UHPLC system (Thermo Fisher Scientific) coupled to a Q Exactive mass spectrometer (Thermo Fisher Scientific). 10 uL injections of the samples were resolved across a 2.1 x 100 mm, 1.7 um particle size Acquity UPLC BEH column (Waters) using a 7 minute, reverse-phase gradient. The mobile phases utilized were

20:80:0.02 acetonitrile:water:formic acid (v:v:v) and 20:80:0.02 acetonitrile:isopropyl alcohol:formic acid (v:v:v). The Q Exactive scanned in negative ion, full MS mode from 150-1500 m/z at 70,000 resolution. The method employed 45 Arb sheath gas, 15 Arb auxiliary gas, and 4 kV spray voltage. Calibration was performed prior to the run using the PierceTM Negative Ion Calibration Solution (Thermo Fisher Scientific). Raw files were converted to .mzXML using RawConverter. Run order of samples was randomized and technical replicates were injected regularly to assess quality control. The resultant files were processed with El-Maven (Elucidata) alongside the KEGG database for metabolite assignment and peak integration as previously described.

Mass Spectrometry-Based Metal Analysis

Prior to ICP-MS analysis, 10 µL of RBCs or supernatant were aliquoted into a 15 mL conical tube. 200 µL of 65% nitric acid and 20 ng/mL of gold were added into each sample followed by an addition of 100 µL of 30% hydrogen peroxide and brief vortexing. Samples were then incubated in an oven at 70°C for approximately 2 h. Following incubation, 2190 µL of MilliQ water was added to each tube (final nitric acid percentage of ~5%) and all samples were vortexed briefly. All samples were then diluted 1:15 in a solution consisting of 20 ng/mL and 5% nitric acid. Final dilutions of 1:250 and 1:3750 were then analyzed *via* ICP-MS. Different dilutions were used to ensure all analytes fell within the calibration curves. All chemicals and materials used for ICP-MS analysis were obtained from Thermo Fisher and all ICP-MS calibrants and solutions were obtained from SPEX CertiPrep. An internal standard mix of Bi, Ge, In, Li^6, Lu, Rh, Sc, and Tb was prepared from a 100 µg/mL pre-purchased stock to a final concentration of 10 ppb. This mix was continuously flowed into the system throughout the run to account for instrument drift. Calibration curves were prepared for each analyte that was monitored during the run: ^{23}Na, ^{24}Mg, ^{39}K, ^{44}Ca, ^{57}Fe, ^{63}Cu. Calibration curves for Sodium, Potassium, and Calcium were 5-point curves prepared at 50, 75, 100, 500, and 1000 ppb. The calibration curves for Iron, Copper, and Magnesium were 8-point curves prepared at 1, 4, 10, 50, 75, 100, 500, and 1000 ppb.

Inductively Coupled Plasma Mass Spectrometry Instrumentation

All samples were analyzed on a Thermo Scientific iCAP RQ ICP-MS coupled to a ESI SC-4DX FAST autosampler system utilizing a peristaltic pump. The optimization of the system was

performed before the run by first calibrating with ICP-MS iCAP Q/Qnova Calibration Solution, Specpure. The system was subsequently tuned using a tuning solution consisting of Ba, Bi, Ce, Co, In, Li, and U at 1.00 ± 0.05 µg/L. To monitor performance while the system was running, we continually pumped internal standard mix *via* the peristaltic pump and monitored signal throughout the run.

Macrophage Isolation and Erythrophagocytosis

The liver and spleen tissues were incubated at 37C with agitation for 30 minutes. After incubation, 100 uL of 0.1 M EDTA was added to the tissue-containing tubes and placed on ice. A single cell suspension was created by addition of Hanks buffered salt solution (HBSS, Corning, product #MT21022CV**),** passing through a 100-um filter, and collected in a 15 mL conical tube. The cell suspension was spun at 500g for 5 minutes and the supernatant discarded. The remaining tissue/cell solution was resuspended in 5ml of RBC lysis buffer (Invitrogen eBiosciences, product #00-4333-57), incubated at room temperature for 15 minutes, and centrifuged at 500g for 5 minutes, this step was repeated if RBC presence was sustained. Next, the cells were washed with Miltenyi buffer (HBSS, 0.5 M EDTA, Fetal bovine serum), centrifuged at 500g for 5 minutes, supernatant removed, and resuspended in 7 mL of D10 media (DMEM ThermoFisher A4192101, 10% FBS ThermoFisher 26140079, 1% Pen/Strep ThermoFisher 10378016) and counted. $10x10^7$ cells were seeded on a 24 well plate (ThermoFisher 142475) and incubated for 2 hours at 37C. During the incubation, whole blood was spun down and RBCs isolated from the plasma. RBCs were then opsonized by incubating RBCs with rabbit, anti-mouse RBC IgG at 0.5 mg/mL (Rockland Immunochemicals, Limerick, PA, United States) for 2 hr at room temperature on a shaker. RBCs were washed with HBSS and 10% FBS to remove any remaining Ig antibody. Plated splenic cells were washed twice with HBSS to ensure macrophages were adherent to the plate. Next, splenic cells were co-cultured with opsonized RBCs at a ratio of 1:4 (splenic macrophages:RBCs). Co-culture continued overnight in standard cell culture conditions. The following morning, plates were washed twice with HBSS to remove RBCs and media. 100 uL of trypsin (ThermoFisher 15400054) was added to each well for ~5 minutes at room temperature to remove the adherent cells. After confirming the cells had been removed from the plate via microscopy, 400 uL of D10 media was added to each well to stop trypsinization. Each sample was then moved to a 2 mL Eppendorf tube,

centrifuged at 500g for 5 minutes, supernatant removed, and immediately flash frozen in liquid nitrogen.

Scanning Electron Microscopy

Whole blood samples were prepared for scanning electron microscopy (SEM) according to standard procedures. Briefly, poly-l lysine coated cover slips were covered with 2-3 drops of blood and fixed in 2.5% glutaraldehyde for 20 minutes. Following a buffer rinse, the blood was fixed again in 1% osmium tetroxide for 20 minutes. The cover slips were rinsed with buffer and dehydrated through a graded ethanol series (50%, 70%, 90%, 100%-X2) for 10 minutes each, then immediately critical point dried using a Leica CPD300 (Buffalo Grove, IL) Critical Point Dryer. The coverslips were then mounted to SEM stubs and sputter coated for 1 min using a gold/palladium target in a Leica EM ACE200 Vacuum Coater. Scanning electron micrographs were acquired using a JEOL (Peabody, MA) JSM-6010LA electron microscope operated in high-vacuum mode at 15kV. Ref: Leica EM CPD300 Application Booklet, Version 05/14. Copyright © by Leica Mikrosysteme GmbH, Vienna, Austria, 2014. RBC quantification was performed using ImageJ and the analysist was blinded to study groups.
